# Comparative transcriptome analysis of resistant and susceptible Kentucky bluegrass varieties in response to powdery mildew infection

**DOI:** 10.1186/s12870-022-03883-4

**Published:** 2022-11-02

**Authors:** Yujuan Zhang, Wenke Dong, Chunxu Zhao, Huiling Ma

**Affiliations:** Key Laboratory of Grassland Ecosystem of Ministry of Education, College of Grassland Science, Gansu Agricultural University, Lanzhou, 730070 China

**Keywords:** *Poa pratensis*, Powdery mildew, Transcriptome, Carbohydrate metabolism, Physiological changes

## Abstract

**Background:**

*Poa pratensis* is one of the most common cold-season turfgrasses used for urban turf building, and it is also widely used in ecological environment management worldwide. Powdery mildew is a common disease of *P. pratensis*. To scientifically and ecologically control lawn powdery mildew, the molecular mechanism underlying the response of *P. pratensis* to powdery mildew infection must better understood.

**Results:**

To explore molecular mechanism underlying the response of *P. pratensis* to powdery mildew infection, this study compared physiological changes and transcriptomic level differences between the highly resistant variety ‘BlackJack’ and the extremely susceptible variety ‘EverGlade’ under powdery mildew infection conditions. We analyzed DEGs using reference canonical pathways in the Kyoto Encyclopedia of Genes and Genomes (KEGG) database, and the results showed that “starch and sucrose metabolism”, “photosynthesis” and “fatty acid metabolism”pathways were only enriched in ‘BlackJack’, and the expression of DEGs such as *HXK*, *INV*, *GS*, *SS*, *AGpase* and *β-amylase* in “starch and sucrose metabolism” pathway of ‘BlackJack’ were closely related to powdery mildew resistance. Meanwhile, compared with ‘EverGlade’, powdery mildew infection promoted synthesis of sucrose, expression of photosynthesis parameters and photosynthesis-related enzymes in leaves of ‘BlackJack’ and decreased accumulation of monosaccharides such as glucose and fructose.

**Conclusions:**

This study identified the key metabolic pathways of a *P. pratensis* variety with high resistance to powdery mildew infection and explored the differences in physiological characteristics and key genes related to sugar metabolism pathways under powdery mildew stress. These findings provide important insights for studying underlying molecular response mechanism.

**Supplementary Information:**

The online version contains supplementary material available at 10.1186/s12870-022-03883-4.

## Background

*Poa pratensis* is one of the main cold-season turfgrasses used in turf construction and ecological environment management in the world because of its long growth and green period, soft grass quality and bright colour [1]. Powdery mildew is a common polycyclic disease on lawns that can be scattered on the surface of host plants through the air or by wind or insects, then causes widespread epidemics that seriously affect plant health and increase the difficulty of prevention and control [2, 3], and usually occurs in shaded lawn areas and seed fields used for seed production on *P. pratensis* [4]. Moreover, powdery mildew is more likely to occur on bluegrass when the air is not circulating, the relative humidity is increased, the light intensity is low and the temperature ranges from 16 to 22℃ [5]. *Blumeria graminis* is the main pathogen that causes powdery mildew in lawns, and it has a wide range of hosts and mainly damages the entire aboveground organs of grasses [6]. The disease caused by this pathogen has small, nearly circular, white spots on the leaves at the initial stage, gradually expands the grey-brown fluffy mildew or grey-white powdery mildew layer in the later stage, and merges into a sheet after covering the whole plants. All these symptoms seriously affect the photosynthesis of plants, increase the respiration intensity and transpiration rate, and finally cause the leaves to turn yellow [3, 7]. In turf grasses, *B. graminis* can infect a variety of grasses, such as *Cynodon dactylon*, *P. pratensis*, *Fineleaf fescues*, *Agrostis stolonifera* and *Dactylis glomerata*, and it has the most severe effect on *P. pratensis* and *C. dactylon* [8, 9].

As we know, when pathogenic fungi enter plants at early stage, it triggers the basic immune response pattern triggered immunity (PTI) and specific immune response effector triggered immunity (ETI) of plants [10, 11]. Among them, pattern recognition receptors (PRRs) on the surface and intracellular regions of plants trigger PTI by sensing molecular pattern signals related to pathogen infection, thus forming first line of defence against pathogen infection [12], for example, physical changes of stomatal closure, cell wall thickening and callose deposition, as well as the physiological reactions of reactive oxygen species (ROS), plant hormones and signal compounds [13]. Pattern signals include microbe-associated molecular patterns (MAMPs), pathogen-associated molecular patterns (PAMPs) and damage-associated molecular patterns (DAMPs) [14, 15]. Carbohydrates, namely sugars, produced by plants through photosynthesis are not only the main energy source of plants and substrates for the synthesis of other organic matter [16], but also serve as DAMPs to sense pathogen and induce defence responses [17]. Studies have indicated that sucrose stimulated activity of phenylalanine ammonialyase (PAL) enzyme in phenylpropane metabolic pathway when *Fusarium oxysporum* infects lupine [18]; trehalose induced peroxidase (POD) activity, which was involved in wheat resistance to powdery mildew [19]; in addition, sugars could also act as antioxidants to remove excess ROS produced during photosynthesis [20]. In studies of the plants resistance genes expression induced by sugars, Thibaud et al*.* [21] found that sucrose and glucose could activate the expression of pathogenesis-related (PR) protein coding gene *PR-2* in Arabidopsis; Sutton et al*.* [22] found that the increased expression of genes related to monosaccharide vector AtSTP4 in wheat leaves infected with powdery mildew led to the accumulation of glucose content in infected leaves; and Moore et al*.* [23] found that the gene *Lr67* encoding hexose transporter (LR67res) reduced glucose uptake of pathogenic bacteria by reducing glucose content of resistant wheat, thereby imparting partial resistance to rust and powdery mildew. In addition, pentose phosphate, tricarboxylic acid cycle, glycolysis and other key pathways in response to diseases resistance mechanism of pathogen need sugars a link with carbon metabolism system [24]. Certainly, the immune mechanism of plants against pathogen is a complex and efficient process. Although many studies have revealed the relationship between metabolic pathways, genes and diseases resistance mechanisms, different species may respond differently to such mechanisms; therefore, further investigations are required.

Transcriptomics has been used to find key genes in recent years, proteins or metabolites related to resistance in plants, and findings have revealed new methods of studying the molecular mechanisms plants response to pathogen infection and resistance breeding [25]. Using comparative transcriptomics, many DEGs related to disease resistance have been identified in different species, such as gerbera [26], gastrodia [27] and cucumber [28]. In addition, transcriptomics has also been used to identify powdery mildew resistance genes in other gramineous plants, including maize [29], wheat [30] and barley [31], as well as key genes and metabolic pathways of plant responses to pathogen infection, thus providing a theoretical basis for revealing the mechanism underlying plants disease resistance response. For example, Wu et al*.* [32] used transcriptome sequencing technology to examine transcriptome expression profile of grape infected by downy mildew, and obtained 15,249 potential DEGs, most of which were involved in sugar metabolism, photosynthesis, amino acid metabolism and other related pathways.

Therefore, to study mechanism underlying the response of *P. pratensis* with different resistances under powdery mildew infection, reveal the molecular mechanism of *P. pratensis* disease resistance at the transcriptome level, and deeply analyse the physiological response related to the sugar metabolism pathway related to disease resistance, this study used the Illumina HiSeq 4000 platform [33] to conduct a transcriptome sequencing analysis of the highly resistant variety ‘BlackJack’ and the extremely susceptible variety ‘EverGlade’ under powdery mildew infection conditions. The results provide a new reference for breeding of *P. pratensis* that is resistant to powdery mildew as well as for the control of lawn powdery mildew.

## Results

### Evaluation of disease resistance of 10 varieties of *P. pratensis*

**Fig. 1:**
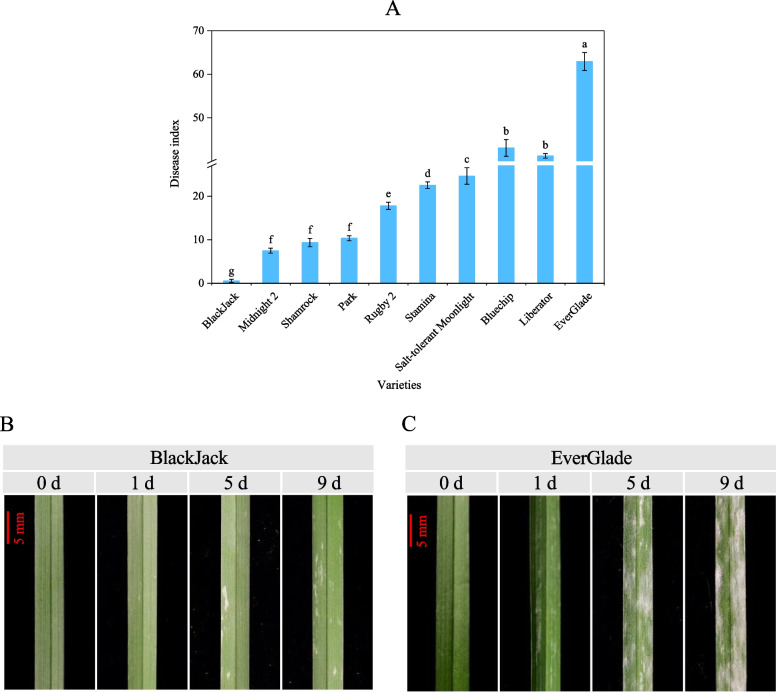
Evaluation of disease resistance of 10 varieties of *P. pratensis*. (**A**) The disease index of 10 *P. pratensis*. Disease severity of ‘BlackJack’ (**B**) and ‘EverGlade’ (**C**) at different time after powdery mildew infection. All data are presented as means ± SE from three independent experimental replicates, different letters indicate significantly different at *P* < 0.05

Disease index is an important tool for determining disease resistance in different varieties [34]. After inoculation with powdery mildew pathogen, the disease index of 10 regularly used *P. pratensis* cultivars was between 0.55 and 62.93 (Fig. 1A). Among them, the ‘BlackJack’ disease index had the lowest of 0.55, indicating excellent disease resistance, while ‘EverGlade’ had the highest disease index of 62.93, showing the worst resistance to pathogen (Fig. 1A). White colonies on the leaves of ‘BlackJack’ became obvious on the 5th day of powdery mildew infection, and gradually developed on the 9th day, but remained at a low level (Fig. 1B). In contrast, large colonies appeared on the 1st day of infection of ‘EverGlade’, and disease spots expanded and gradually formed white powder layer on the 5th day. On the 9th day, spores eventually developed and began to form yellow–brown to black closed capsules (Fig. 1C). Therefore, ‘BlackJack’ was established as a highly resistant variety to powdery mildew while ‘EverGlade’ was established as a susceptible variety.

### Physiological indexes of two *P. pratensis* varieties after powdery mildew infection

**Fig. 2:**
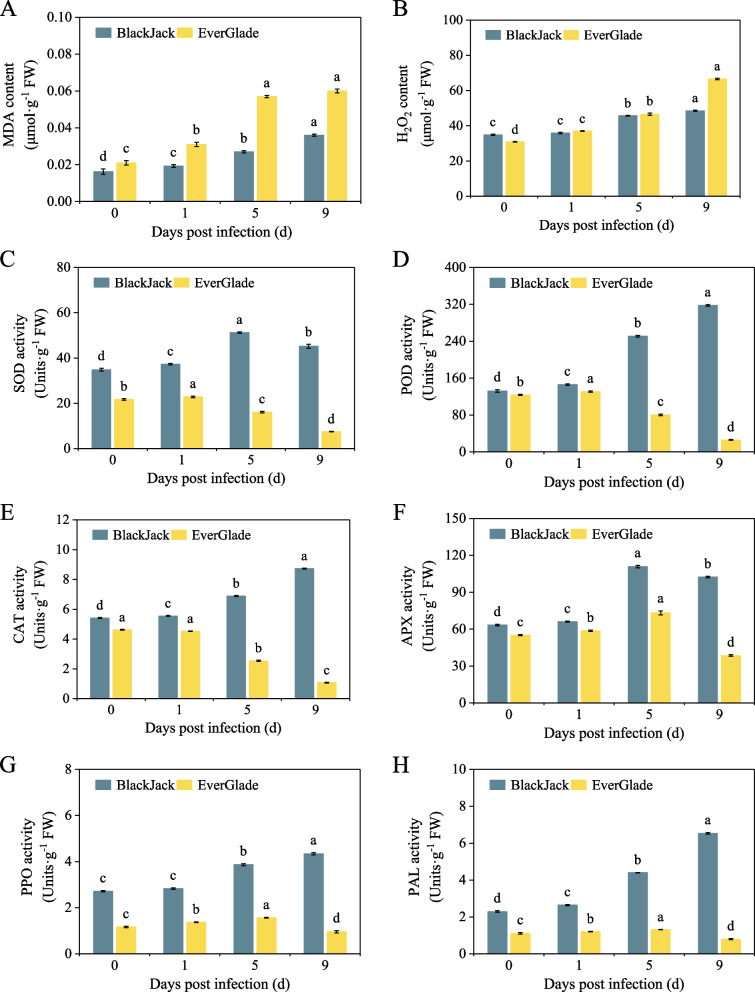
Effect of powdery mildew infection on physiological indexes of two *P. pratensis* seedlings. The MDA (**A**), H_2_O_2_ (**B**), SOD (**C**), POD (**D**), CAT (**E**), APX (**F**), PPO (**G**), and PAL (**H**) activity were measured. All data are presented as means ± SE from three independent experimental replicates, different letters indicate significantly different at *P* < 0.05, the same below

Malondialdehyde (MDA) and hydrogen peroxide (H_2_O_2_) are major ROS involved in plants and pathogen interaction and significant indicators of plants oxidative damage caused by pathogen [35]. The results of this study showed that as the period of powdery mildew infection increased, the MDA and H_2_O_2_ contents of ‘BlackJack’ and ‘EverGlade’ increased as well, reaching a maximum on the 9th day (Fig. 2A and B). During period of inoculation, the MDA and H_2_O_2_ contents of ‘BlackJack’ were lower than those of ‘EverGlade’, but the H_2_O_2_ content of ‘BlackJack’ did not vary significantly between the 0th day and 1st day (*P* > 0.05), and was also lower than that of ‘EverGlade’ on the 0th day (Fig. 2A and B).

Superoxide dismutase (SOD), peroxidase (POD), hydroperoxidase (CAT) and ascorbate peroxidase (APX) together constitute the antioxidant system of plants to remove excess ROS under stress conditions [36]. With the increase of pathogen infection time, the SOD and APX activities of ‘BlackJack’ initially increased and then decreased, reached a maximum on the 5th day after inoculation, and were significantly higher on the 9th day than 0th day (Fig. 2C and F). The activity of POD and CAT increased as inoculation time increased and showed a gradual upward trend in ‘BlackJack’ (Fig. 2D and E). Conversely, the SOD and POD activities in ‘EverGlade’ decreased significantly after reaching a maximum on the 1st day and reached a minimum on the 9th day (Fig. 2C and D). Moreover, the CAT activity of the ‘EverGlade’ seedlings was not significantly difference between the 0th day and 1st day after infection and then decreased significantly, reaching the minimum value on the 9th day, which was 0.2 times that of the 0th day (Fig. 2E). Although the APX activity of ‘EverGlade’ reached the maximum value on the 5th day, it still decreased to 0.7 times on the 9th day that of the 0th day (Fig. 2F). The activity of antioxidant enzymes in ‘BlackJack’ was higher than that in ‘EverGlade’ both uninoculated and inoculated infection plants (Fig. 2C, D, E and F).

PAL and polyphenol oxidase (PPO) are two key enzymes in the phenylpropane metabolic pathway that contribute to the production of lignin and phenolic substances and play an important role in plants disease resistance [37]. With the increase of powdery mildew infection time, the PPO and PAL activities of ‘BlackJack’ seedlings increased gradually and reached the maximum increases of 59.8% and 184.6% on the 9th day, respectively, compared with the uninoculated control; however, significant differences were not observed in PPO activity on the 0th and 1st days (Fig. 2G and H). The PPO and PAL activities of ‘EverGlade’ seedlings increased initially and then decreased with inoculation time, reaching a maximum increase of 34.3% and 18.3% on the 5th day, respectively, compared with the control. Throughout the treatment period, the activities of PPO and PAL in the ‘BlackJack’ seedlings were consistently higher than those in the ‘EverGlade’ seedlings (Fig. 2G and H). These physiological indexes showed that ‘BlackJack’ had better disease resistance than ‘EverGlade’ (Fig. 2).

### Statistics of transcriptome sequencing and assembly

To completely analyse the transcriptome and gene expression profiles of ‘BlackJack’ and ‘EverGlade’ under different treatments, 12 cDNA samples were extracted from the leaves and sequenced using the Illumina HiSeq 4000 platform (San Diego, CA, USA). Each sample was sequenced to generate more than 39 million raw and clean reads and more than 5.80 G total bases. Furthermore, the base error rate was at a low level of 0.02%, the Q20 value was greater than 95.00%, and the Q30 value was greater than 89.00%. The GC content was between 54.72% and 57.61%. A total of 602,015,640 raw reads were obtained from 12 samples (Additional file 1: Table S1). After excluding reads with linkers and low-quality reads and ensuring that proportion of unknown nucleotides was no more than 10%, a total of 597,710,016 clean reads were obtained, with a total size of bases was 88.52 G (Additional file 1: Table S1). clean data were de novo assembled by Trinity software, and the results were evaluated. A total of 420,875 unigenes were obtained from 12 samples. The average length of the unigenes was 545.17 bp and the N50 length was 684 bp (Additional file 2: Table S2). Furthermore, for a total of 286,069 unigenes, the length was mostly distributed between 200 and 500 bp (Additional file 2: Table S2).

### Functional annotation and classification of unigenes

To comprehensively analyse and predict the function of unigenes, all unigenes obtained by transcriptome assembly were annotated using BLAST and four major databases (NR, Swiss-Prot, KEGG and COG). A total of 168,148 (39.95%) of the 420,875 unigenes could be significantly matched in at least one of the four databases. Among them, 164,756 (39.15%), 94,859 (22.54%), 32,454 (7.71%) and 71,588 (17.01%) unigenes were significantly matched to the NR, Swiss-Prot, KEGG and COG databases, respectively (Additional file 3: Figure S1).

The results of unigenes functional annotation in NR database showed that the species that could be compared with *P. pratensis* were primarily *Brachypodium distachyon* (25.14%), *Hordeum vulgare* subsp. Vulgare (18.31%), *Aegilops tauschii* (15.55%), *Triticum urartu* (11.62%),*Triticum aestivum* (5.04%), *Oryza sativa* Japonica Group (4.48%), *Setaria italica* (2.55%), *Zea mays* (2.06%) and Sorghum bicolor (1.89%) (Additional file 4: Figure S2).

GO annotation analysis was performed to classify the predicted functions of *P. pratensis* unigenes. A total of 248,240 unigenes were successfully annotated and classified into three main categories, including molecular functions (91,966), cellular components (67,771) and biological processes (88,503), the top three terms in the molecular functions category were “catalytic activity (40,870, 16.46%)”, “binding (40,071, 16.14%)” and “transporter activity (3,887, 1.57%)”; in the cellular components category were “cell part (22,261, 8.97%)”, “organelle (17,393, 7.01%)” and “membrane (9,078, 3.66%)”; and in the biological processes category were “metabolic process (32,068, 12.92%)”, “cellular process (22,836, 9.20%)” and “single-organism process (11,906, 4.80%)” (Additional file 5: Figure S3A).

To further predict gene function and evaluate the integrity of the *P. pratensis* transcriptome, all unigenes were examined in the COG database. Based on this database, 71,588 unigenes were classified into 25 functional categories. Among these categories, the largest category was “signal transduction mechanisms (12,217, 17.07%)”, followed by “posttranslational modification, protein turnover, chaperones (9,050, 12.64%)” and “general function prediction only (8,978, 12.54%)”; the smallest category was “cell motility (21, 0.03%)”, followed by “extracellular structures (247, 0.35%)” and “nuclear structure (241, 0.34%)” (Additional file 5: Figure S3B).

The unigenes obtained for *P. pratensis* were annotated in the KEGG metabolic pathway, and a total of 73,978 unigenes were assigned to 135 metabolic pathways. The 135 annotated pathways could be divided into six primary pathways: “cellular process (2,624)”, “environmental information processing (2,187)”, and “genetic information processing (12,985)”, “human diseases (2)”, “metabolism (54,397)” and “organismal systems (1,783)”. The six primary pathways could be further divided into 20 secondary pathways, of which the number of enriched genes was large, and five of these secondary pathways were “global and overview maps (26,265)” and “carbohydrate metabolism (7,867)”, “translation (5,235)”, “amino acid metabolism (4,790)” and “folding, sorting and degradation (4,619)” (Additional file 5: Figure S3C).

### Statistics and analysis of DEGs

**Fig. 3:**
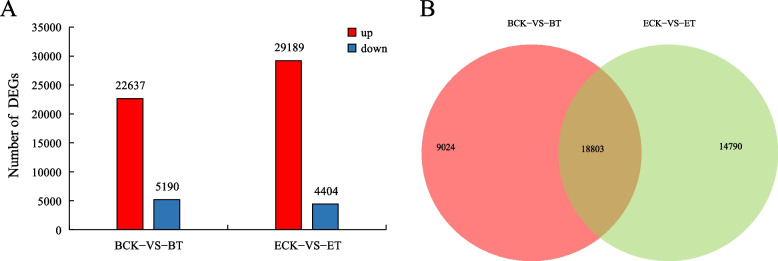
Statistical analysis of DEGs in response to powdery mildew infection in *P. pratensis*. (**A**) The numbers of upregulated and downregulated DEGs among the two ‘BlackJack’ and ‘EverGlade’; (**B**) Venn diagram showing transcripts distribution in ‘BlackJack’ and ‘EverGlade’. ‘BlackJack’ control group (**BCK**), ‘BlackJack’ treatment group (**BT**), ‘EverGlade’ control group (**ECK**) and ‘EverGlade’ treatment group (**ET**)

The genes expression of the two *P. pratensi* varieties changed considerably after inoculation with powdery mildew as compared to the control, with |log_2_(fold change)|≥ 2 and FDR ≤ 0.05 used as the standard. However, the number of modified genes was different, ‘BlackJack’ included 22,637 up-regulated DEGs and 5,190 down-regulated DEGs, whereas ‘EverGlade’ included 29,189 up-regulated and 4,404 down-regulated (Fig. 3A). The number of DEGs obtained by comparing the two varieties were counted, and the Venn diagram was utilized to demonstrate the unique and common DEGs of each species. The results revealed that 18,803 DEGs were shared by two species, 9,024 DEGs were unique to ‘BlackJack’ and 14,790 DEGs were unique to ‘EverGlade’ (Fig. 3B).

### GO functional enrichment analysis of DEGs

**Fig. 4:**
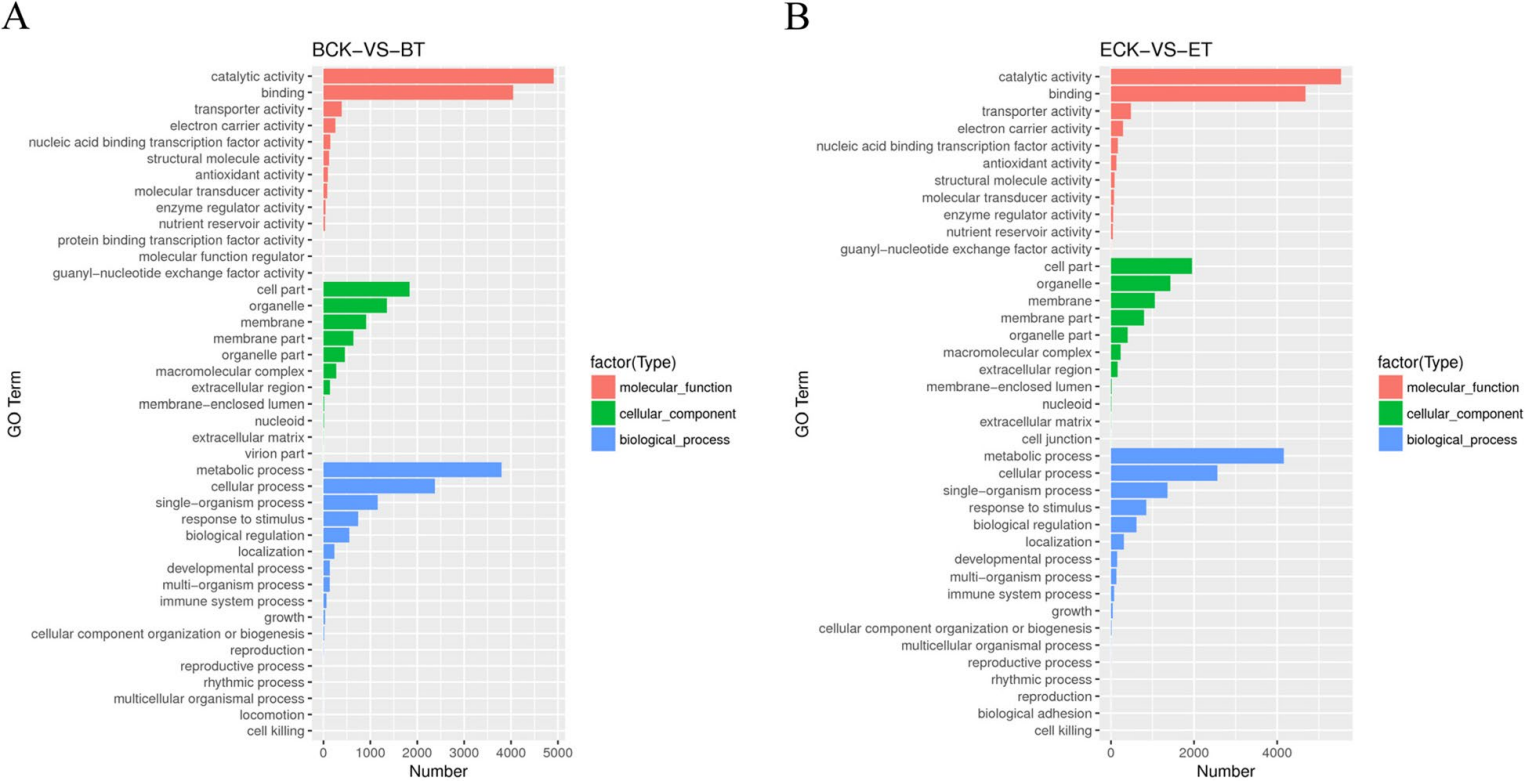
The GO enrichment analysis of DEGs of ‘BlackJack’ (**A**) and ‘EverGlade’ (**B**) with differents resistance to powdery mildew infection. ‘BlackJack’ control group (**BCK**), ‘BlackJack’ treatment group (**BT**), ‘EverGlade’ control group (**ECK**) and ‘EverGlade’ treatment group (**ET**)

GO enrichment analysis was performed for the DEGs of ‘BlackJack’ and ‘EverGlade’, and the results showed that all DEGs were classified into three categories, including “molecular functions”, “cellular components” and “biological processes” (Fig. 4). Based on the corrected *P* value, we selected the 30 most enriched GO entries. In the molecular functions, the main terms were “catalytic activity” and “binding”, in the cell components were “cell part”, “organelle” and “membrane”, in the biological processes were “metabolic process” and “single-organism process” of two varieties (Fig. 4A and B).

### KEGG enrichment analysis pathway of DEGs

**Fig. 5:**
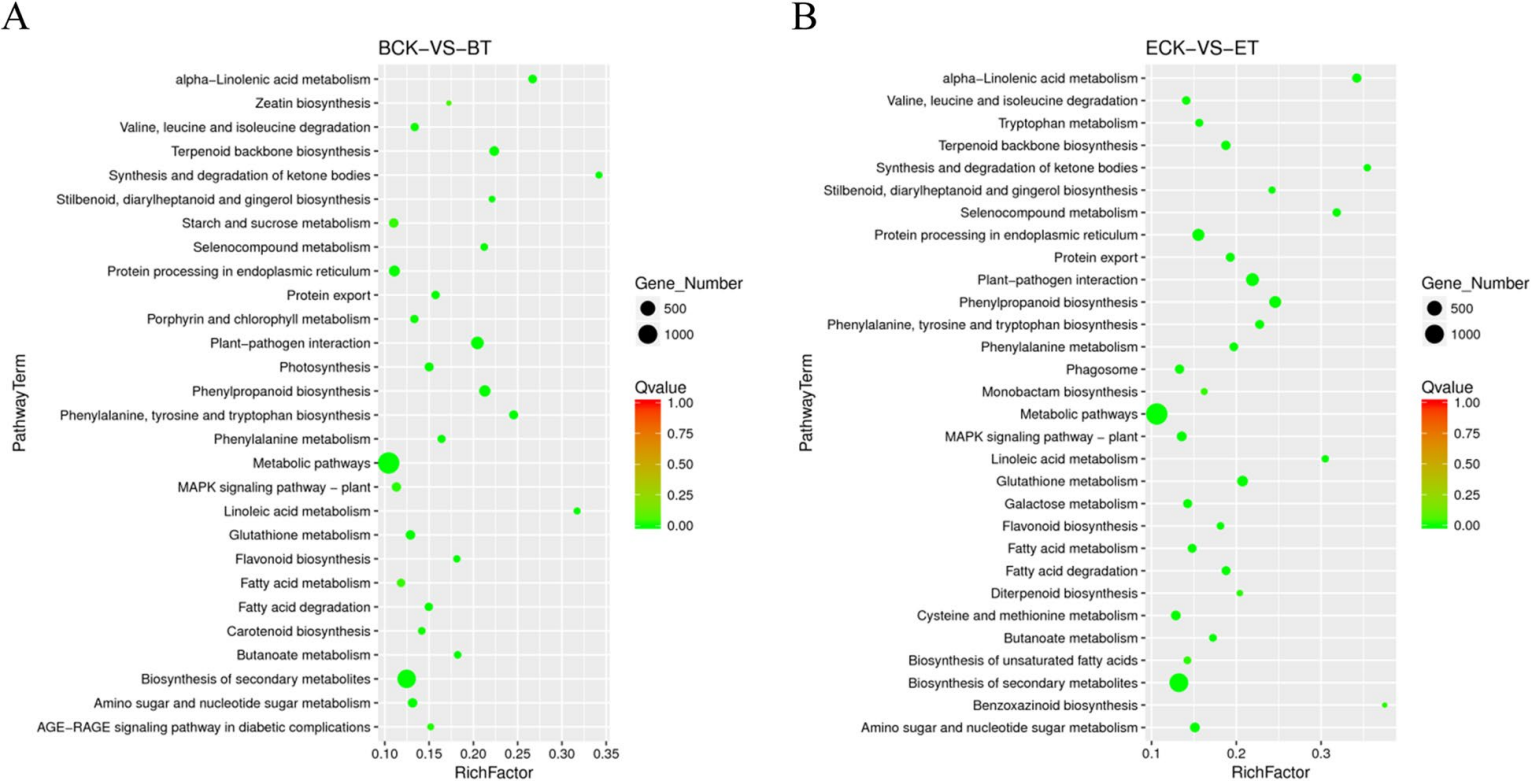
The KEGG pathway enrichment analysis of DEGs of ‘BlackJack’ (**A**) and ‘EverGlade’ (**B**) with differents resistance to powdery mildew infection. ‘BlackJack’ control group (**BCK**), ‘BlackJack’ treatment group (**BT**), ‘EverGlade’ control group (**ECK**) and ‘EverGlade’ treatment group (**ET**)

The DEGs were mapped to canonical pathways in KEGG database. Based on the number of enriched DEGs, we identified the top 30 pathways (Fig. 5). In ‘BlackJack’, the most abundant KEGG pathways were “metabolic pathways (ko01100)”, “biosynthesis of secondary metabolites (ko01110)”, “plant-pathogen interaction (ko04626)”, “phenylpropanoid biosynthesis (ko00940)”, “protein processing in endoplasmic reticulum (ko04141)”, “terpenoid backbone biosynthesis (ko00900)”, “glutathione metabolism (ko00480)”, “starch and sucrose metabolism (ko00500)”, “amino sugar and nucleotide sugar metabolism (ko00520)” and “MAPK signaling pathway-plant (ko04016)” (Fig. 5A). In ‘EverGlade’, the most abundant KEGG pathways were “metabolic pathways (ko01100)”, “biosynthesis of secondary metabolites (ko01110)”, “plant-pathogen interactions (ko04626)”, “protein processing in endoplasmic reticulum (ko04141)”, “phenylpropanoid biosynthesis (ko00940)”, “glutathione metabolism (ko00480)”, “amino sugar and nucleotide sugar metabolism (ko00520)”, “MAPK signaling pathway-plant (ko04016)”, “cysteine and methionine metabolism (ko00270)” and “terpenoid backbone biosynthesis (ko00900)” (Fig. 5B).

### DEGs involved in starch and sucrose metabolism

Starch and sucrose metabolism plays a key role in regulating the interaction between plants and pathogen. In this study, when powdery mildew infected two varieties, the DEGs of starch and sucrose metabolism were only significantly enriched in ‘BlackJack’. The KEGG pathway analysis revealed that a total of 102 (3.63%) DEGs in ‘Black Jack’ were annotated to starch and sucrose metabolism pathways. Among them, the DEGs encoding hexokinase (HXK, EC: 2.7.1.1), invertase (INV, EC: 3.2.1.26), glucose-6-phosphate isomerase (PGI, EC: 5.3.1.9), glycogen phosphorylase (GS, EC: 2.4.1.1), glycogen (starch) synthase (GP, EC: 2.4.1.11) and alpha,alpha-trehalase (THL, EC: 3.2.1.28) were significantly up-regulated while those encoding ADP-glucose synthase (AGP, EC: 2.7.7.27), starch synthase (SS, EC: 2.4.1.21) and beta-amylase (BMY, EC: 3.2.1.2) were down-regulated (Additional file 6: Figure S4).

### DEGs expression and carbohydrate associated with starch and sucrose metabolism

**Fig. 6:**
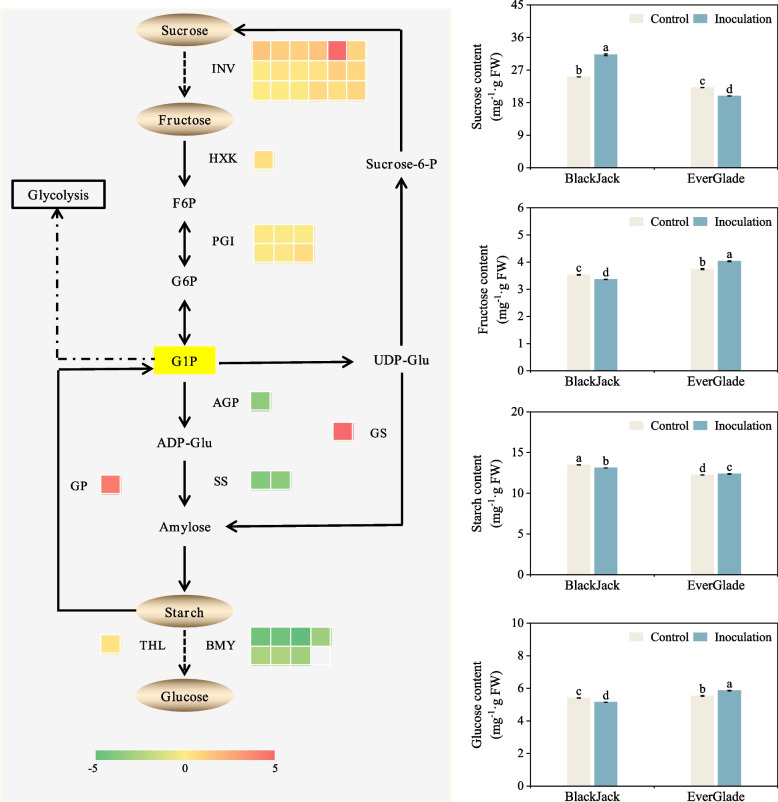
Schematic diagram of starch and sucrose metabolism of ‘BlackJack’ and carbohydrate content. Colour gradients from green to red represents the Log_2_FC of DEGs in heat maps. All data are presented as means ± SE from three independent experimental replicates, different letters indicate significantly different at *P* < 0.05

Heatmaps of the key DEGs in starch and sucrose metabolism were constructed, and the contents of the main carbohydrates in this pathway were analysed. The sucrose content of ‘BlackJack’ seedlings in the pathway of starch and sucrose metabolism increased significantly after powdery mildew infection and was significantly higher than that of the control and ‘EverGlade’ (*P* < 0.05). At the same time, 25 genes encoding INV, HXK and PGI were up-regulated throughout the transformation from sucrose to fructose and then to glucose-1-phosphate (G1P), and the fructose content in ‘BlackJack’ decreased by 4.7% compared with that of control. In transformation of glucose-1-phosphate to starch, the genes related to AGP and SS were down-regulated in the process of ADP-glucose, although genes related to GS were up-regulated in the process of UDP-glucose. Moreover, the genes related to THL were up-regulated in the process of starch decomposition into glucose, but 7 genes connected to BMY were down-regulated, and another gene related to the enzyme GP was up-regulated in the process of starch decomposition into G1P. Compared to the control, the starch and glucose contents of ‘BlackJack’ seedlings decreased while the glucose content of ‘EverGlade’ seedlings increased by 5.8% and the glucose content of ‘EverGlade’ was significantly higher than that of ‘BlackJack’ before and after infection (*P* < 0.05) (Fig. 6).

### qRT-PCR verification of transcriptome analysis

**Fig. 7:**
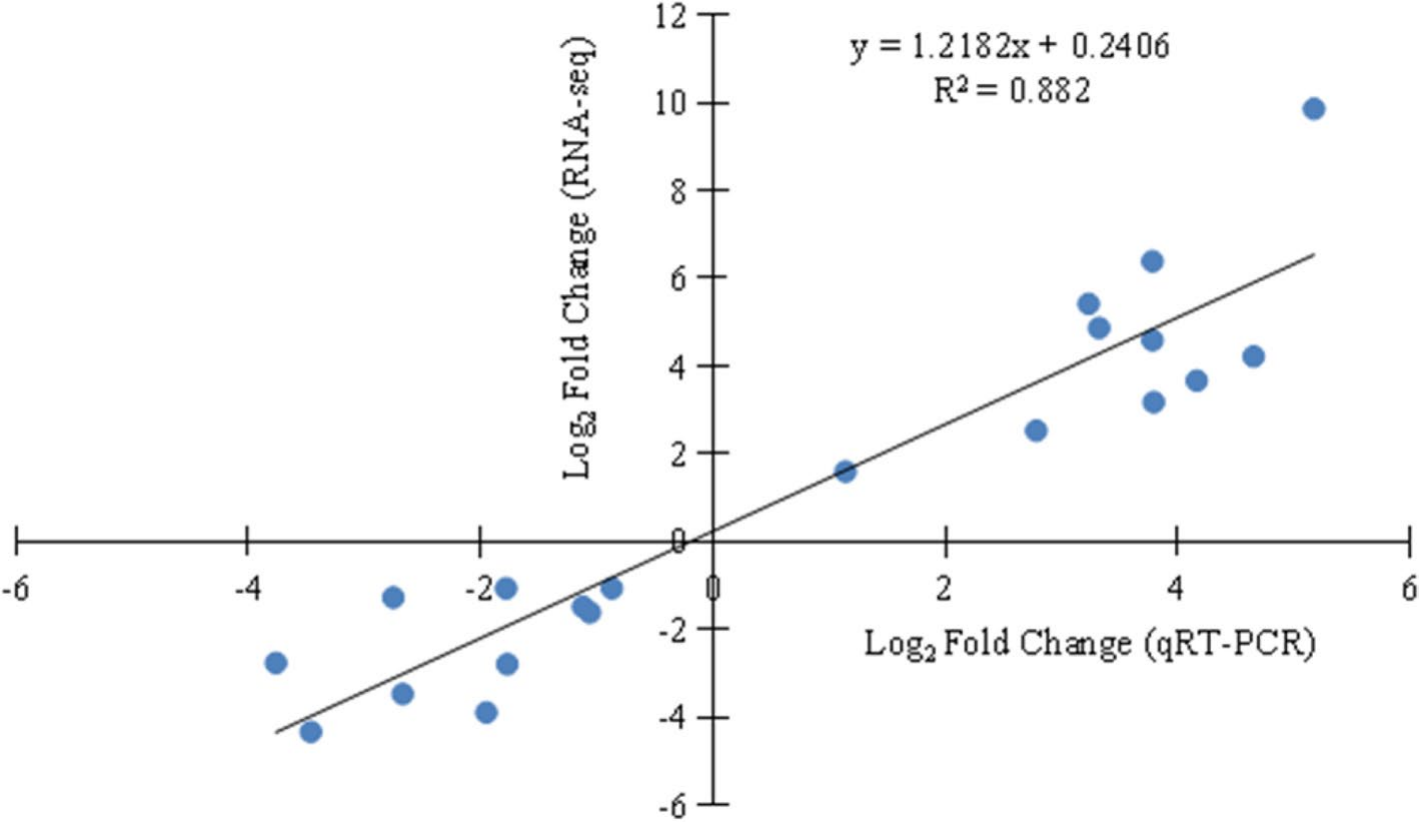
Correlation of RNA-seq and qRT-PCR date

To further verify the reliability of the data, we randomly selected 20 DEGs for qRT-PCR. The qRT-PCR results were basically consistent with the RNA-Seq data, and the correlation coefficient (R^2^) reached 0.882, confirming the reliability of RNA-Seq data (Fig. 7 and Additional file 7: Table S3).

### Effect of powdery mildew infection on photosynthetic characteristics and related enzymes of *P. pratensis*

**Fig. 8:**
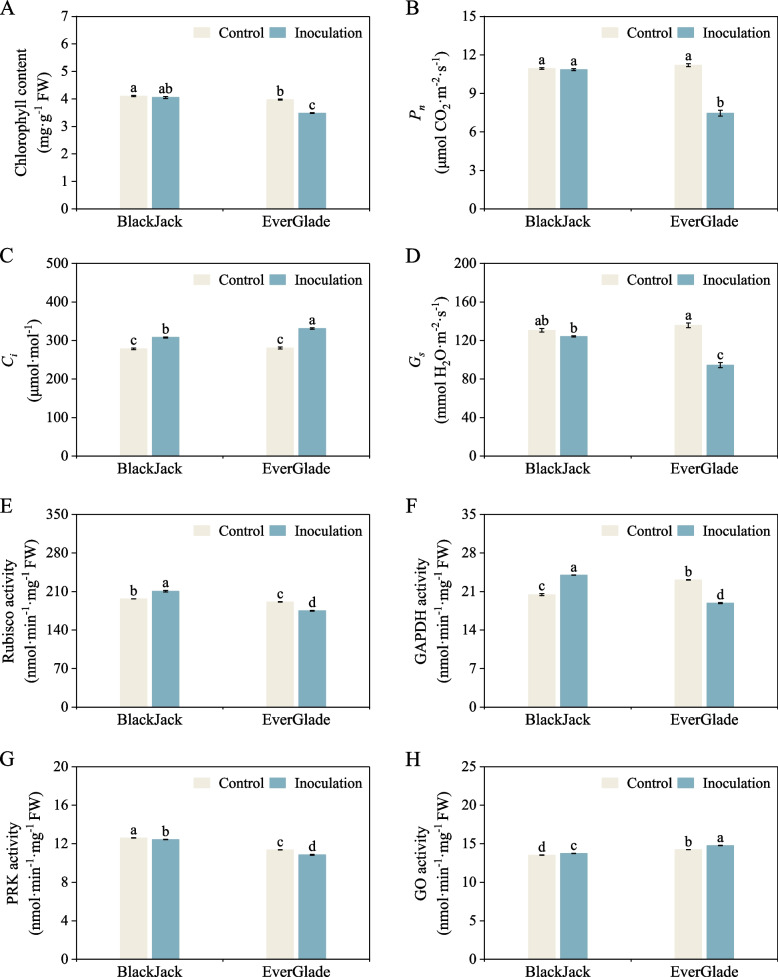
Effect of powdery mildew infection on photosynthetic characteristics and related enzymes of *P. pratensis*. Chlorophyll (**A**), *P*_*n*_ (**B**), *C*_*i*_ (**C**), and *G*_*s*_ (**D**), Rubisco (**E**), GAPDH (**F**), PRK (**G**) and GO (**H**) activity were measured. All data are presented as means ± SE from three independent experimental replicates. Different letters indicate significantly different at *P* < 0.05

Chlorophyll has a direct impact on the photosynthetic capacity of plants and the synthesis or accumulation of organic matter in photosynthetic organs [38]. After inoculation, the chlorophyll content of ‘BlackJack’ seedlings was less affected by pathogen and differed significantly relative to the control without inoculation (*P* > 0.05), but after infection by pathogen, the chlorophyll content of ‘EverGlade’ decreased significantly to 87.6% of the control (Fig. 8A). Net photosynthetic rate (*P*_*n*_), intercellular CO_2_ concentration (*C*_*i*_) and stomatal conductance (*G*_*s*_) are main parameters that stimulate the photosynthetic immune defence response during pathogen infection [39]. In this study, significant differences were not observed in the *P*_*n*_ and *G*_*s*_ of ‘BlackJack’ before and after infection (*P* > 0.05), while those of ‘EverGlade’ decreased significantly after inoculation and were both lower than those of ‘BlackJack’ (Fig. 8B and D). The *C*_*i*_ of the varieties showed an upward trend after infection, and the increase was even greater in ‘EverGlade’ (Fig. 8C). Ribulose-1,5-bisphosphate carboxylase (Rubisco), glyceraldehyde-3-phosphate dehydrogenase (GAPDH), phosphoribulokinase (PRK) and glycolate oxidase (GO) are important photosynthetic carbon cycle enzymes that are essential for the synthesis of sucrose and other organic substances [40, 41]. In this study, after infection, the activity of Rubisco and GAPDH increased in ‘BlackJack’ but the activity in ‘EverGlade’ decreased significantly (Fig. 8E and F). At the same time, the PRK activity of ‘BlackJack’ and ‘EverGlade’ decreased after infection while the GO activity increased and was significantly higher in ‘EverGlade’, before and after infection (*P* < 0.05) (Fig. 8G and H).

## Discussion

*B. graminis* is one of the main diseases that threaten the establishment of turfgrass [2, 3]. Zhu et al*.* [6] showed that the bluegrass leaves had a large amount of white cover about 12 days under powdery mildew infection conditions. This study found the same phenomenon occurred in the susceptible variety on the 9 day of powdery mildew pathogen infection. Li et al*.* [30] found that the pathogen colonized in wheat leaves 24 h after inoculation of powdery mildew. This study also found that two varieties had the phenomenon of pathogenic fungi colonization on the 1st day, and it was more serious in the susceptible variety. The reason may be that the environment conducive to the growth of powdery mildew caused the short incubation period of the disease [5, 42]. Plants present a series of defence mechanisms and promote the expression of defence genes to resist pathogen invasion [12]. In this study, the changes of basic physiological indexes and related enzyme activities of two bluegrass varieties after powdery mildew pathogen infection further indicated that ‘BlackJack’ had better disease resistance than ‘EverGlade’, and there was significant difference between the two varieties and their control (the 0th day), also had an excellent difference between the resistant and susceptible varieties on the 5th day (Fig. 2). RNA-Seq transcriptome sequencing technology represents a comprehensive and accurate method for studying potential genes and gene regulatory networks mediated by plants response to abiotic stress [43]. Wan et al*.* [44] determined the time of transcriptome sampling by observing the colonization of spores in leaves and analyzed the disease resistance in combination with the physiological changes of leaves during this time period. Considering the time specificity of transcriptome analysis, in this study, we used RNA-Seq sequencing technology, and selected selected the leaves without pathogen inoculation as the control group and the leaves on the 5th day after inoculation as the treatment group, in order to analyze the molecular mechanisms underlying the responses of two bluegrasses to powdery mildew infection at the physiological and transcriptomic levels and identify the metabolic pathways and genes associated with disease resistance.

Sugars are essential for plants growth and serve as carbon precursors in the Calvin cycle and carbon metabolism, glycolysis, and other pathways [16]. When exposed to biological stress, sugars can also activate signal transduction in metabolic pathways and stimulate the expression of a range of defence genes [45, 46]. Studies have pointed out that metabolic pathways associated with sugars are the key to control the defence response of plants to fungal pathogen pathogen [47]. Fang et al*.* [48] found that many DEGs in the resistant variety were primarily connected to carbohydrate metabolism, photosynthesis and secondary metabolites when studying the anthracnose of walnut caused by *Colletotrichum gloeosporioides*. In this study, KEGG was used to analyze the DEGs of ‘BlackJack’ and ‘EverGlade’, and results showed that the DEGs involved in the “starch and sucrose metabolism”, “photosynthesis” and “fatty acid metabolism” pathways were only significantly enriched in ‘BlackJack, with 102 DEGs annotated to “starch and sucrose metabolism” (Fig. 5), which is essential for plants growth, stress response and yield formation [49, 50].

Hexokinase (HXK, EC: 2.7.1.1), invertase (INV, EC: 3.2.1.26) (also known as β-fructofuranosidase), glucose-6-phosphate isomerase (PGI, EC: 5.3.1.9) and glycogen (starch) synthase (GP, EC: 2.4.1.11) are key catalytic enzymes in the pathway of starch and sucrose metabolism. Sucrose must be phosphorylated by HXK to produce most glucose and fructose [51]. HXK is active in transducing sugar signals, sensing biotic and abiotic stress, and regulating plant genes expression and growth [52]. It is found that HXK mainly exists as a multi-gene family in monocots, such as 10 OsHXKs have been identified in rice [53]. Wang et al*.* [54] found that overexpression of *OsHXK1* in rice induced the production of ROS and enhanced the resistance of rice to RBSDV infection. Qi et al*.* [55] showed that low concentrations of ROS could activate the expression of defense related genes and induce plants defense response, while high levels of ROS would cause oxidative damage to plant cells. This study found that the MDA and H_2_O_2_ contents of ‘BlackJack’ increased slightly after pathogen infection compared to the control, whereas that of ‘EverGlade’ increased dramatically (Fig. 2A and B). Therefore, this phenomenon may also be directly involved in the resistance of two bluegrass varieties. INV catalyses the dismutation of sucrose into fructose and is crucial in the distribution of sucrose. Currently, genes encoding INV have been identified from a variety of plants, such as *Atβfruct* in arabidopsis [56], *InvDC* in carrot [57], and *Ivrl* in corn [58]. Glycogen phosphorylase (GS, EC: 2.4.1.1) is a major enzyme for synthesizing plants starch, with UDP-glucose as the sugar donor in sucrose and starch metabolism [59]. Our results indicated that in starch and sucrose metabolism, both intracellular and extracellular sucrose in the process of forming glucose-1-phosphate up-regulated the expression of HXK, INV and PGI (Fig. 6). The formed glucose-1-phosphate has three destinations: the first is to synthesize starch through ADP-glucose, the second is to synthesize sucrose or starch through UDP-glucose, and the third is to provide products for glycolysis. However, this study also found that the genes expression of GS were up-regulated in the process of starch synthesis through UDP-glucose, while the genes expression of ADP-glucose synthase (AGP, EC: 2.7.7.27) and starch synthase (SS, EC: 2.4.1.21) were down-regulated in the ADP-glucose to starch pathway (Fig. 6). Previous research showed that plants mainly used ADP-glucose as a synthesis substrate during starch synthesis, followed by UDP-glucose [60]. Fan et al*.* [61] pointed out that the starch metabolism of susceptible variety was disordered after pathogen infection and massive accumulation of starch led to damage to the chloroplast thylakoid system. Johnson et al*.* [62] found that starch accumulation in susceptible citrus leaves led to phloem tissue blockage and necrosis. This study found that the starch content of ‘BlackJack’ decreased slightly after pathogen infection, while that of ‘EverGlade’ increased (Fig. 6). Therefore, in this study, ‘BlackJack’ showed resistance to the disease, which may be due to the down-regulation of related enzymes in the pathway of starch synthesis with ADP-glucose as the main glycosyl donor, resulting in the failure of starch accumulation (Fig. 6). Moreover, glucose-1-phosphate was synthesized by up-regulating glycogen phosphorylase (GP, EC: 2.4.1.1) to maintain starch metabolism in ‘BlackJack’ within a certain balanced range. Therefore, the excessive accumulation of starch may be an important feature of the susceptible bluegrass variety. This study also found that 7 genes associated with beta-amylase (BMY, EC: 3.2.1.2) were down-regulated and one gene associated with alpha,alpha-trehalase (THL, EC: 3.2.1.28) was up-regulated during starch decomposition into glucose. Among them, BMY is an exo-amylase that hydrolyses starch successively from the non-reducing end, and it is essential for grasses to decompose starch into glucose [63, 64]. Relevant studies have shown that sugars are not only necessary for plants growth, but also a nutrient source for pathogenic fungi. Pathogenic fungi use sugars for their growth, however, in most cases, pathogenic fungi cannot directly use sucrose and starch, which must be decomposed into monosaccharides such as glucose and fructose [47, 65]. Therefore, fructose and glucose are regarded as essential regulators of plant defensive responses against fungal infection [66]. This study found that after powdery mildew infection, the contents of glucose and fructose in ‘BlackJack’ decreased, while the contents in ‘EverGlade’ increased (Fig. 6). Therefore, the disease resistance of ‘BlackJack’ in this study may also be because the down-regulation of BMY related genes reduced the accumulation of glucose, while the up-regulated expression of enzymes HXK and PGI that catalyzed fructose decomposition, led to the decrease in fructose content, resulting in the decrease in the available monosaccharides content of pathogen and thus inhibiting the growth. But the increase of monosaccharides content in the susceptible variety provided nutrients for the expansion of pathogens, which may be an important reason for the rapid propagation of powdery mildew in the later stage (Fig. 6). In addition, several up-regulated genes in this study were involved in the synthesis of glucose-1-phosphate, which is an essential regulatory point in glycolysis [67], therefore, the disease resistance of ‘BlackJack’ may also be related to the participation of glucose-1-phosphate in the glycolytic pathway.

In plants, photosynthesis occurs in the chloroplast, ATP and NADPH are produced through photoreactions in the chloroplast matrix and used as the energy required for the Calvin Cycle in carbon fixation reactions to complete the conversion of CO_2_ reduction to triose [68]. Triose can be transported to the cytoplasm through the “phosphate translocator” on the inner envelope to synthesize sucrose or starch in the interstitium of the chloroplast, and starch is temporarily stored in amyloplasts during the day and decomposed with glucose as a signal at night [69]. Sucrose is synthesized through photosynthesis, as the main form of organic matter distribution, and it completes the transportation of sugar from source to sink through plasmodesmata or cell wall to regulate plants growth, development and stress response, with starch and sucrose metabolism playing a role in this process [45]. Chlorophyll is an essential index for measuring the photosynthetic capacity of plants. Balachandran et al*.* [70] found that pathogen infection could lead to the degradation of chlorophyll in leaves. This study also found that the chlorophyll content in ‘BlackJack’ decreased after the pathogen infection, but not significantly compared to the control, while that of ‘EverGlade’ decreased significantly (Fig. 8A).

Yang et al*.* [39] showed that when photosynthesis provides signals and substances for plant immune defence, the immune defence process could also have a feedback effect on photosynthesis,and the most direct effect was a change in the photosynthetic parameters *P*_*n*_, *G*_*s*_ and *C*_*i*_. The factors that reduce the photosynthetic rate of plants caused by pathogen infection are stomatal limitation caused by stomatal closure or non-stomatal limitation caused by the decline of photosynthetic activity of plants, *P*_*n*_, *G*_*s*_ and *C*_*i*_ are the basis for judging stomatal or non-stomatal limitation. When *C*_*i*_ and *G*_*s*_ decrease at the same time, the decrease of *P*_*n*_ is a stomatal limitation; on the contrary, when *G*_*s*_ decreases but *C*_*i*_ increases, the decrease in photosynthesis is a non-stomatal limitation [71]. Our study showed that the *P*_*n*_ and *G*_*s*_ of leaves of the two bluegrass cultivars decreased to varying degrees after inoculation with powdery mildew, while *C*_*i*_ showed an upward trend (Fig. 8B, C and D), indicating that the decline in the photosynthetic rate of bluegrass after powdery mildew infection was caused by non-stomatal limitation. At the same time, the photosynthetic parameters of ‘BlackJack’ were less affected by powdery mildew than those of ‘EverGlade’, and the photosynthetic rate and stomatal conductance remained relatively stable in ‘BlackJack’ (Fig. 8B, C and D), thus providing favourable conditions for the subsequent photosynthesis. Rubisco enzyme is essential in the process of photosynthetic carbon assimilation, and its activity has a direct effect on the photosynthetic rate of plants infected by pathogen [72]. GAPDH enzyme can utilize the reducing force generated by photosynthesis to catalyse the conversion of primary photosynthetic products in the Calvin cycle [73]. In this study, these two enzymes increased in ‘BlackJack’ but decreased in ‘EverGlade’ significantly (Fig. 8E and F), therefore, this phenomenon may also ensure the carbon assimilation ability and stability of synthetic photosynthetic products of the resistant variety during pathogen infection. PRK is a unique enzyme of the Calvin cycle that regulates sugar flow and interacts with GAPDH for oxidative stress in plants [74, 75]. We found that the PRK activity of two bluegrass varieties showed a downward trend after infection (Fig. 8G). The reason for this might be that PRK is less susceptible to oxides than GAPDH [74], although the decline in ‘BlackJack’ was minimal (Fig. 8G), which also provided a driving force for the generation and transportation of photosynthetic substrates. Go enzyme is a hinge in the process of plants photorespiration, which catalyses the oxidation of glycolic acid to glyoxylic acid and produces an equal molar amount of H_2_O_2_, which is closely related to the induction of plant disease resistance [76]. Study have shown that when plants were infected by pathogen, GO activity was significantly affected; for example, when barley was infected by *Bipolaris sorokiniana*, GO activity would increase [77]. This study also found that after powdery mildew infection, GO activity increased in both resistant and susceptible varieties (Fig. 8H), ensuring the consistency between the two bluegrass varieties and GO enzyme related processes during pathogen infection.

However, combined with chlorophyll, relevant indicators of photosynthetic parameters and key enzymes activity of Calvin cycle, the photosynthetic capacity of the disease resistant variety ‘BlackJack’ in this study was better than that of ‘EverGlade’, which also explained why the sucrose content of the resistant and susceptible bluegrass cultivars showed an opposite trend after inoculation with powdery mildew, namely, the sucrose content of ‘BlackJack’ showed significant upward trend while that of ‘EverGlade’ showed a significant decrease (Fig. 6). In conclusion, these results suggested that resistant variety could respond to powdery mildew infection by regulating genes expression associated with starch and sucrose metabolism induced by photosynthesis.

## Conclusions

The above-mentioned results indicate that a large number of DEGs were specifically expressed in the “starch and sucrose metabolism” pathway in the resistant variety in response to powdery mildew infection, and may affect the substrate needed in this pathway through regulating photosynthesis, thus leading to the enhancement of resistance to powdery mildew in the variety. However, after the susceptible variety was infected by powdery mildew, no DEGs were enriched in the “starch and sucrose metabolism” pathway and the photosynthetic capacity decreased, which provided an opportunity for the further expansion of the pathogen. Although more studies are needed to discover the mechanism underlying *P. pratensis* resistance to powdery mildew, this study has preliminarily established a pattern expression map of the “starch and sucrose metabolism” pathway and identified many potential genes for disease resistance breeding, thus providing guidance for the innovation of *P. pratensis* germplasm.

## Methods

### Plants and pathogen materials

The seeds of 10 common varieties of *P. pratensis* (‘Park’, ‘Shamrock’, ‘BlackJack’, ‘Midnight 2’, ‘Rugby 2’, ‘Stamina’, ‘EverGlade’, ‘Liberator’, ‘Salt-tolerant Moonlight’ and ‘Bluechip’) were used as experimental materials. The seeds were provided by the Beijing Clover Grass Technology Development Centre (Beijing, China) and stored in its herbarium. *B. graminis* was isolated from the leaves of diseased *P. pratensis* in lawn training at the Grass Industry College of Gansu Agricultural University and was identified by the College of Plant Protection of Gansu Agricultural University. Spores on the leaves of *P. pratensis* infested with powdery mildew were collected in the field and gently brushed into sterile water to make a mother liquor. The spore concentration was calculated with a haemocytometer. The mother liquor was made into a spore suspension with sterile water so that the concentration of the spore suspension was 1 × 10^6^ spores·mL^−1^, and 0.1% Tween-20 was added for the inoculation test.

### Inoculation of pathogenic spore suspension

The prepared spore suspension was sprayed evenly on the leaves of the *P. pratensis* seedlings until all the leaves had the suspension and made it dripped naturally as a liquid when powdery mildew inoculated. Each group received approximately 50 mL, with clean water as a control, and the relative humidity of the cultivation environment was maintained at about 60% during the onset of disease.

### Seeds treatment and seedlings cultivation

Ten varieties seeds of *P. pratensis* with full particles and uniform size were selected and evenly sown in a nursery bowl after disinfection with 20% NaClO. The substrate in the nursery bowl was nutritious soil (enriched soil:vermiculite = 2:1, the composition of enriched soil were garden soil, humus and plant ash by Golden Land Biotechnology Co., Ltd. (SuQian, China)), which was sterilized under high temperature and high pressure. After sowing, the seeds were grown in an artificial climate chamber at Gansu Agricultural University, Gansu Lanzhou in China. The growth conditions were as follows: the day and night temperature was 25 ± 1℃; the light/dark cycle was 16 h/8 h; the light intensity was 5000 Lux; and the humidity was 50%-70%. When the seedlings of *P. pratensis* reached approximately 10 cm and reached 50 plants per pot, 10 pots of seedlings were taken as a group, 1 biological replicate was set for 3 groups, and 3 biological replicates were set for each varieties.

### Evaluation of disease resistance in 10 *P. pratensis* varieties

The disease index of ‘Park’, ‘Shamrock’, ‘BlackJack’, ‘Midnight 2’, ‘Rugby 2’, ‘Stamina’, ‘EverGlade’, ‘Liberator’, ‘Salt-tolerant Moonlight’ and ‘Bluechip’ seedlings were calculated on the 9th day under powdery mildew infection, 30 seedlings were randomly selected from each replicate, and 3 biological replicates were set up. Referring to the “Rules investigation and forecast for wheat powdery mildew (*B. graminis* (DC.) Speer)” (NY/T613-2002) and the grading standards of Zhang et al. [78], the grading standard of *P. pratensis* powdery mildew was determined as follows:

0: no symptoms; 1: a small amount of fine and fuzzy white spots and lesion area accounts for less than 5% of the whole leaf; 3: thin powdery layer and diseased area accounts for 6–10% of the whole leaf; 5: thick white powder layer and lesion area accounts for 11–20% of the whole leaf; 7: thick white powder layer and the lesion area accounts for 21–40% of the whole leaf; 9: relatively thick white powder layer and lesion area accounts for more than 40% of the whole leaf. The calculation formulas for the disease index are as follows:

$$Disease\;index (DI) = (\sum NDL \times GLDS)/(TNIL\times\;THGL) \times 100,$$

where NDL is the number of diseased leaves at each level; GLDS is the grade level of disease severity; TNIL is the total number of investigated leaves; and THGL is the highest-grade level.

The grading criteria for resistance were as follows: DI = 0, immunity (I); 0 < DI ≤ 5.0, high resistance (HR); 5.0 < DI ≤ 15.0, middle resistance (MR); 15.0 < DI ≤ 25.0, middle susceptibility (MS); 25.0 < DI ≤ 50.0, high susceptibility (HS); and DI > 50.0, extremely susceptibility (ES).

### Effect of powdery mildew infection on the physiological characteristics of *P. pratensis*

Based on the previous disease resistance evaluation, the high powdery mildew resistant variety ‘BlackJack’ and the extremely susceptible powdery mildew variety ‘EverGlade’ were used test materials. Subsequently, 1.0 g of leaves were randomly collected from each replicate at 0, 1, 5, and 9 days after inoculation, and each index included 3 biological replicates, quickly frozen in liquid nitrogen and stored in an ultra-low temperature refrigerator at -80℃ for the analysis of physiological and biochemical indexes. The MDA content was determined by thiobarbituric acid (TBA) method [79]. The H_2_O_2_ content was determined by UV spectrophotometer [80]. The SOD activity was measured by nitroblue tetrazolium chromogenic method [81]; The POD activity was measured by guaiacol method [82]; The CAT activity was determined by UV colorimetry [83]. APX activity was measured based on the method of Nakano et al. [84]. PPO activity was determined according to the method of Dalton et al. [85]. PAL activity referred to the method of Ruiz et al. [86]. The experiment included 3 biological replicates.

### Transcriptomics analysis

Referred to the method of Wan et al*.* [44], we combined the greater differences in the phenotypes and physiological indexes of two varieties to determine the time point for transcriptome analysis on the 5th day after inoculation, randomly collected 1.0 g leaves from the resistant variety ‘BlackJack’ and the susceptible variety ‘EverGlade’, and used the water treatment without inoculation treatment in the same period as the control, each variety was set with 3 biological replicates. They were labeled as ‘BlackJack’ control group (BCK), ‘BlackJack’ treatment group (BT), ‘EverGlade’ control group (ECK) and ‘EverGlade’ treatment group (ET).

### RNA extraction and quality control

Total RNA was extracted from the collected *P. pratensis* leaves using an extraction reagent (Tiangen Biotech, Beijing, China). The degradation and contamination of the extracted RNA were evaluated based on 1.0% agarose electrophoresis. RNA purity was evaluated by an Ultramicro spectrophotometer (SpectraMax® QuickDrop™, Shanghai, China), and only RNA samples with A260/A280 in the range of 1.8–2.0 were used for the subsequent analyses. The RNA integrity of the samples was evaluated with an Agilent 2100 bioanalyzer (Agilent, Palo Alto, MA), and only the samples with RNA integrity number (RIN) in the range of 8–10 were further analysed.

### cRNA library construction and Illumina sequencing

After qualifying the RNA samples, Oligo (dT) and poly magnetic beads were used to perform A-T base matching on the mRNA enriched in eukaryotes, and fragmentation buffer was added to break the mRNA into short fragments. Based on the short mRNA fragments, under the action of reverse transcriptase, first-strand cDNA was synthesized with six-base random primers, and then double-strand cDNA was purified with AMPure XP beads. The purified double-stranded cDNA was first added to End Repair Mix for end repair; then adaptor ligation was carried out after the addition of the ‘A’ tail; and AMPure XP beads were used for selecting the fragment size. Finally, PCR was performed for 15 cycles. The PCR products were recovered by 2.0% agarose electrophoresis, and they were purified by AMPure XP beads to obtain the final cDNA library. After the library passed the quality inspection, Illumina HiSeq sequencing was performed by GENEWIZ (Suzhou, China).

### Transcriptome assembly and gene functional annotation

The raw data obtained by Illumina high-throughput sequencing were converted into text signals by CASAVA base recognition and then stored as raw reads in fastq format. Clean reads were obtained by performing quality control on the raw reads by removing reads containing adaptors, ‘N’ (‘N’ represents information that cannot determine the base) and low-quality reads (> 50% of the bases with a Q-value ≤ 5) [87]. Since the sequencing information of the whole genome of *P. pratensis* has not yet been published, clean reads were de novo assembled by Trinity software (v 2.90) in this study [88]. The longest transcript from the spliced transcript sequence was selected as the ‘unigene’, and all transcripts and unigenes were analysed, and used for subsequent biological information analysis. The BLASTx algorithm was used to compare the transcripts obtained by transcriptome sequencing and unigenes were assessed based on five major databases [89]: the Non-Redundant Protein Sequence Database (NR), Annotated Protein Sequence Database (Swiss-Prot), Cluster of Orthologous Groups of proteins (COG), Gene Ontology (GO) and Kyoto Encyclopaedia of Genes and Genomes (KEGG), to obtain annotation information for each library. The BLASTx (v 2.2.28 +) method (E-value ≤ 10^–5^) was used to divide unigenes into single genes and families (similar rate > 80%). The sequence direction was determined according to the best hit in the database.

### In-depth analysis of DEGs

The transcriptome sequences acquired by Trinity were used as the reference sequence, RSEM software (v 1.3.1) was used to map the clean reads of each sample to the reference sequence, and FPKM conversionwas performed to analyse the expression level of each unigene [90]. NOIseq software (v 1.20.0) was used to analyse the DEGs between the inoculation and control samples, and the default criteria of DEGs were |log_2_(fold change)|≥ 2 and FDR ≤ 0.05. Goatools software (v 1.24.0) was used to perform the GO enrichment analysis on unigenes/transcripts in the gene sets to obtain the main GO functions of the genes. Fisher’s exact test was used to evaluate the significance level of the unigene/transcript enrichment of a GO function term. At a corrected P value < 0.05, the GO function is considered significantly enriched [91]. The R Programming Language was used to write a script to perform KEGG pathway enrichment analyses of the unigenes/transcripts in the gene sets. The calculation principle was the same as the GO function enrichment analysis. At a corrected *P* value < 0.05, the KEGG pathway function was considered significantly enriched [92].

### Real-time quantitative PCR (qRT-PCR) verification

To verify the reliability of the transcriptome data, 20 DEGs were randomly selected for validation by qRT-PCR. Total RNA was extracted from the inoculated and control samples using extraction reagent (Tiangen Biotech, Beijing, China). Reverse transcription of RNA into cDNA was performed using the PrimeScript™ II 1st Strand cDNA Synthesis Kit (Solarbio, Beijing, China). Reverse-transcribed cDNA was used as a template, and the *Actin* gene was used as an internal reference [93]. A Lightcycle 96 Real-Time PCR system (Roche, Basel, Switzerland) was used for real-time fluorescent quantitative analysis, and three biological replicates were performed. The specific primers were designed with Primer6.0 software (Additional file 8: Table S4). The real-time fluorescent quantitative PCR reaction system was 20 µL, and the reaction conditions were as follows: predenaturation at 94℃ for 5 min, denaturation at 95℃ for 15 s and annealing at 60℃ for 30 s for 40 cycles. The relative level of gene expression was calculated using the 2^−∆∆Ct^ method [94].

### Determination of carbohydrate content in starch and sucrose metabolism

The sucrose, fructose and glucose contents were determined according to Pescador et al. [95], with some modifications. In detail, 0.1 g leaf samples were ground with liquid nitrogen, 10 mL ultrapure water was added, and the samples were placed in a water bath at 80℃ for 30 min.After cooling to room temperature, the supernatant was filtered and the above step was repeated once to eliminate the remaining impurities. All supernatants were combined, diluted to 25 mL, and passed through a 0.45 µm filter membrane. The sucrose, fructose and glucose contents were measured with a high performance liquid chromatograph (Agilent Palo Alto, MA) [96], using an Agilent ZORBAX Carbohydrate Analysis column, acetonitrile–water ratio of 75:25, column temperature of 35℃, and mobile phase flow rate of 1 mL·min^−1^. The starch content was measured by the anthrone-sulfuric acid method [96].

### Determination of photosynthesis related indexes and enzymes

The chlorophyll content was determined by ethanol extraction [97]. The *P*_*n*_, *C*_*i*_ and *G*_*s*_ of the leaves were measured by a portable photosynthetic instrument GFS-3000 (WALZ, Nuremey, Germany). Rubisco activity was determined according to the method of Cheng et al. [98]. GAPDH and PRK activity were determined according to the method of Marri e*t al*. [99]. GO activity was determined according to the method of Hall et al. [100].

### Statistical analysis

SPSS 20.0 statistical software (SPSS Inc., Chicago, IL, USA) was used for statistical analysis, one-way ANOVA and Duncan’s test were used to analyze significant differences in physiological parameters, Origin 2021 software (OriginLab., Northampton, USA) was used for drawing.

## Supplementary Information

**Additional file 1: Table S1.** Staistics of transcriptome sequencing and assembly of *Poa pratensis*.

**Additional file 2: Table S2.** Staistics of number and length distribution of unigenes.

**Additional file 3: Figure S1.** Venn diagram of unigenes annotation in different database.

**Additional file 4: Figure S2.** Species distribution of NR annotation results.

**Additional file 5: Figure S3.** Functional annotation of unigenes annotation in three databases. Histogram presentation of GO function (**A**), COG (**B**) and KEGG (**C**) classifications of unigenes.

**Additional file 6: Figure S4.** Effect of powdery mildew infection on starch and sucrose metabolism pathway of *Poa pratensis*. Note: Red background indicates up-regulated expression, blue background indicates down regulated expression, and green background indicates up-regulated expression down regulated expression.

**Additional file 7: Table S3.** The date of RNA-seq and qRT-PCR.

**Additional file 8: Table S4.** Primer sequence information of qRT-PCR.

## Data Availability

Raw Illumina sequence data were deposited in the National Center for Biotechnology Information (NCBI) and be accessed in the sequence read archive (SRA) database (https://www.ncbi.nlm.nih.gov/sra). The accession number is PRJNA852358 (https://www.ncbi.nlm.nih.gov/bioproject/PRJNA852358).

## Abbreviations

KEGG: Kyoto Encyclopedia of Genes and Genomes
DEGs: Differentially expressed genes
PTI: Pattern triggered immunity
ETI: Effector triggered immunity
PRRs: Pattern recognition receptors
ROS: Reactive oxygen species
MAMPs: Microbe-associated molecular patterns
PAMPs: Pathogen-associated molecular patterns
DAMPs: Damage-associated molecular patterns
PAL: Phenylalanine ammoniase
POD: Peroxidase
PR: Pathogenesis-related
MDA: Malondialdehyde
H_2_O_2_: Hydrogen peroxide
SOD: Superoxide dismutase
CAT: Hydroperoxidase
APX: Ascorbate peroxidase
PPO: Polyphenol oxidase
HXK: Hexokinase
INV: Invertase
PGI: Glucose-6-phosphate isomerase
GS: Glycogen phosphorylase
GP: Glycogen (starch) synthase
THL: Alpha,alpha-trehalase
AGP: ADP-glucose synthase
SS: Starch synthase
BMY: Beta-amylase
G1P: Glucose-1-phosphate
*P*_*n*_: Net photosynthetic rate
*C*_*i*_: Intercellular CO_2_ concentration
*G*_*s*_: Stomatal conductance
Rubisco: Rubisco ribulose-1,5-bishosphate carboxylase
GAPDH: Glyceraldehyde-3-phosphate dehydrogenase
PRK: Phosphoribulokinase
GO: Glycolate oxidase
