# Supplementary material for: Comparative transcriptome analysis of resistant and susceptible Kentucky bluegrass varieties in response to powdery mildew infection
Source: BMC Plant Biol. 2022 Nov 2;22:509. doi: 10.1186/s12870-022-03883-4 (PMC9628184; doi:10.1186/s12870-022-03883-4)
Supplement: Supplementary file 1 — Additional file 1: Table S1. Staistics of transcriptome sequencing and assembly of Poa pratensis. [file 12870_2022_3883_MOESM1_ESM.docx]

**Table S1** Staistics of transcriptome sequencing and assembly of *Poa pratensis*

| Sample | Raw reads | Clean reads | Bases | Error rate/% | Q20/% | Q30/% | GC/% | N/ppm |
| --- | --- | --- | --- | --- | --- | --- | --- | --- |
| BCK1 | 39,711,466 | 39,361,574 | 5.83G | 0.02 | 96.13 | 90.00 | 55.43 | 41.60 |
| BCK2 | 51,577,670 | 51,104,986 | 7.59G | 0.02 | 96.53 | 90.97 | 57.61 | 14.93 |
| BCK3 | 60,652,990 | 60,288,408 | 8.69G | 0.02 | 98.40 | 95.32 | 55.41 | 23.61 |
| BT1 | 39,842,902 | 39,472,434 | 5.85G | 0.02 | 95.99 | 89.72 | 54.72 | 41.58 |
| BT2 | 73,206,808 | 72,794,616 | 10.83G | 0.02 | 97.14 | 92.53 | 56.00 | 22.73 |
| BT3 | 47,211,126 | 46,804,866 | 6.95G | 0.02 | 96.65 | 91.18 | 55.86 | 16.07 |
| ECK1 | 44,320,512 | 43,963,380 | 6.53G | 0.02 | 96.46 | 90.67 | 56.90 | 16.72 |
| ECK2 | 47,209,074 | 46,897,478 | 6.97G | 0.02 | 96.24 | 89.96 | 57.12 | 19.44 |
| ECK3 | 54,661,858 | 54,356,650 | 8.09G | 0.02 | 97.18 | 92.58 | 57.26 | 22.79 |
| ET1 | 53,283,016 | 52,808,630 | 7.82G | 0.02 | 96.08 | 89.96 | 53.41 | 41.77 |
| ET2 | 46,622,626 | 46,374,662 | 6.90G | 0.02 | 97.28 | 92.85 | 54.95 | 22.85 |
| ET3 | 43,715,592 | 43,482,332 | 6.47G | 0.02 | 97.19 | 92.65 | 54.90 | 23.15 |
| Total | 602,015,640 | 597,710,016 | 88.52G | — | — | — | — | — |
