# Supplementary material for: Comparative transcriptome analysis of resistant and susceptible Kentucky bluegrass varieties in response to powdery mildew infection
Source: BMC Plant Biol. 2022 Nov 2;22:509. doi: 10.1186/s12870-022-03883-4 (PMC9628184; doi:10.1186/s12870-022-03883-4)
Supplement: Supplementary file 2 — Additional file 2: Table S2. Staistics of number and length distribution of unigenes. [file 12870_2022_3883_MOESM2_ESM.docx]

**Table S2** Staistics of number and length distribution of unigenes

| Type | All_Unigene |
| --- | --- |
| Sequences | 420,875 |
| Minimum length | 201 |
| Maximum length | 15,279 |
| Mean length | 545.17 |
| N50 | 684 |
| <200 | 0 (0.00%) |
| 200-500 | 286,069 (67.97%) |
| 500-1000 | 88,216 (20.96%) |
| 1000-1500 | 24,136 (5.73%) |
| 1500-2000 | 10,524 (2.50%) |
| ≥2000 | 11,929 (2.83%) |
