# Supplementary figures and images for: Comparative transcriptome analysis of resistant and susceptible Kentucky bluegrass varieties in response to powdery mildew infection
Source: BMC Plant Biol. 2022 Nov 2;22:509. doi: 10.1186/s12870-022-03883-4 (PMC9628184; doi:10.1186/s12870-022-03883-4)

**
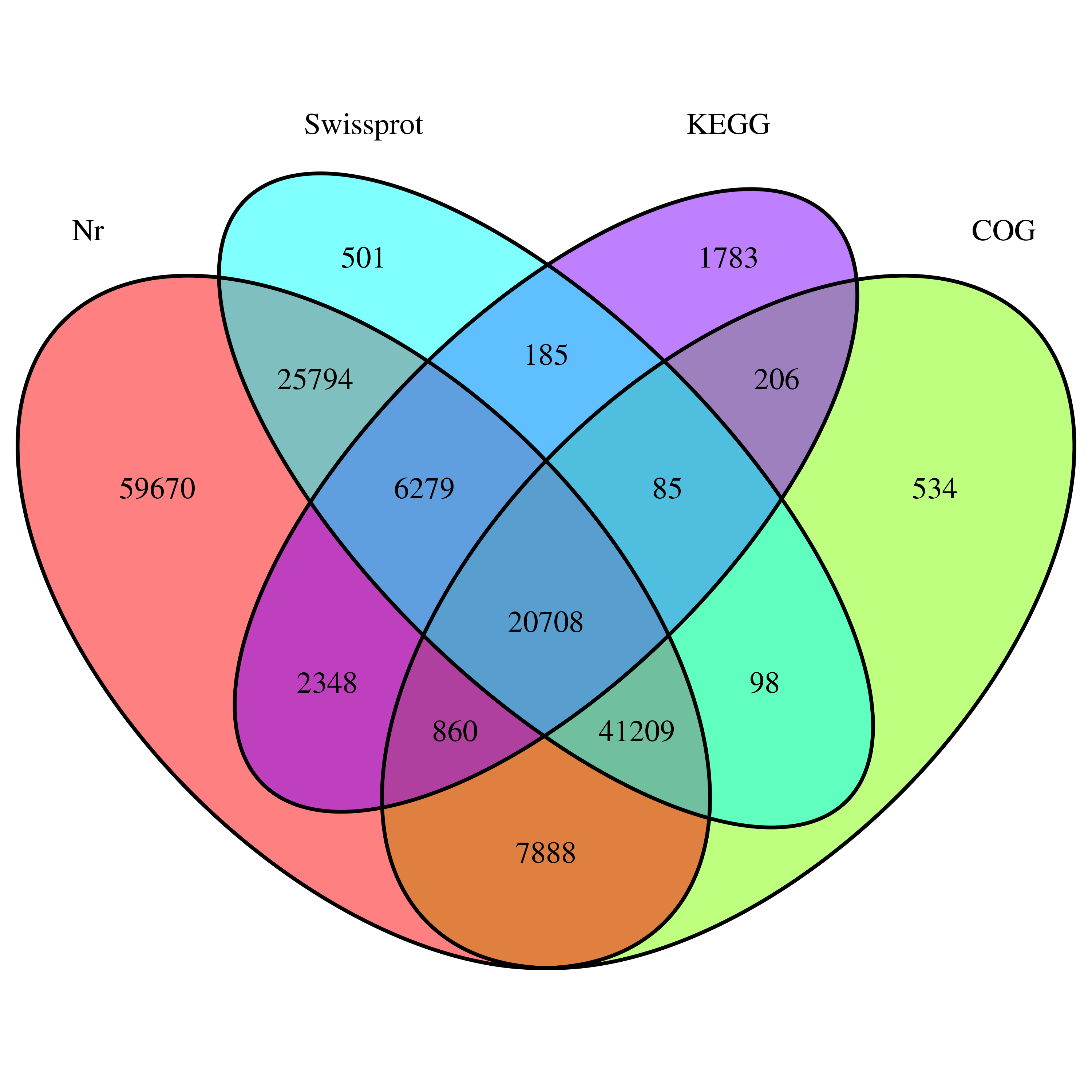
**

**Figure S1.** Venn diagram of unigenes annotation in different database

Supplement: Supplementary file 3 — Additional file 3: Figure S1. Venn diagram of unigenes annotation in different database. [file 12870_2022_3883_MOESM3_ESM.docx]
