# Supplementary material for: Comparative transcriptome analysis of resistant and susceptible Kentucky bluegrass varieties in response to powdery mildew infection
Source: BMC Plant Biol. 2022 Nov 2;22:509. doi: 10.1186/s12870-022-03883-4 (PMC9628184; doi:10.1186/s12870-022-03883-4)
Supplement: Supplementary file 4 — Additional file 4: Figure S2. Species distribution of NR annotation results. [file 12870_2022_3883_MOESM4_ESM.docx]

**Figure S2.** Species distribution of NR annotation results
