# Supplementary material for: Comparative transcriptome analysis of resistant and susceptible Kentucky bluegrass varieties in response to powdery mildew infection
Source: BMC Plant Biol. 2022 Nov 2;22:509. doi: 10.1186/s12870-022-03883-4 (PMC9628184; doi:10.1186/s12870-022-03883-4)
Supplement: Supplementary file 5 — Additional file 5: Figure S3. Functional annotation of unigenes annotation in three databases. Histogram presentation of GO function (A), COG (B) and KEGG (C) classifications of unigenes. [file 12870_2022_3883_MOESM5_ESM.docx]

**
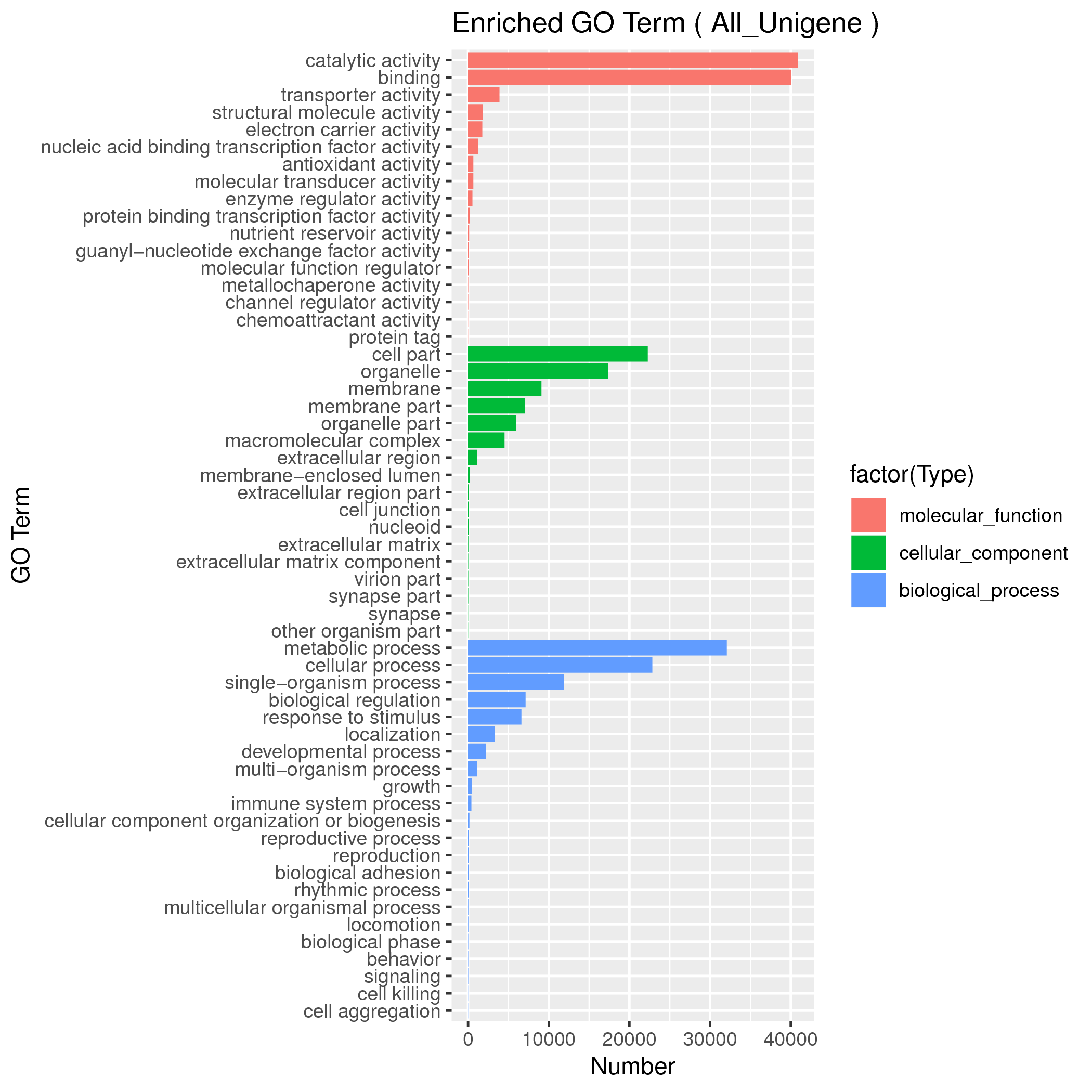
**

A

**
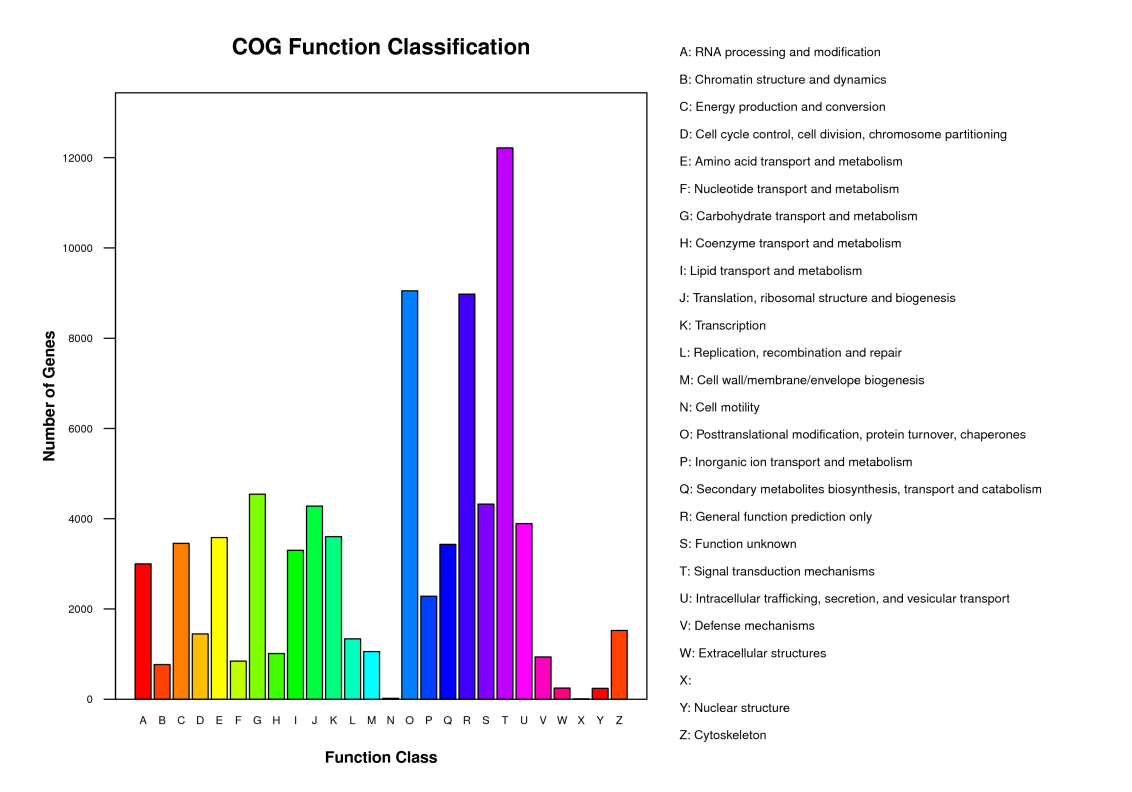
**

B

**
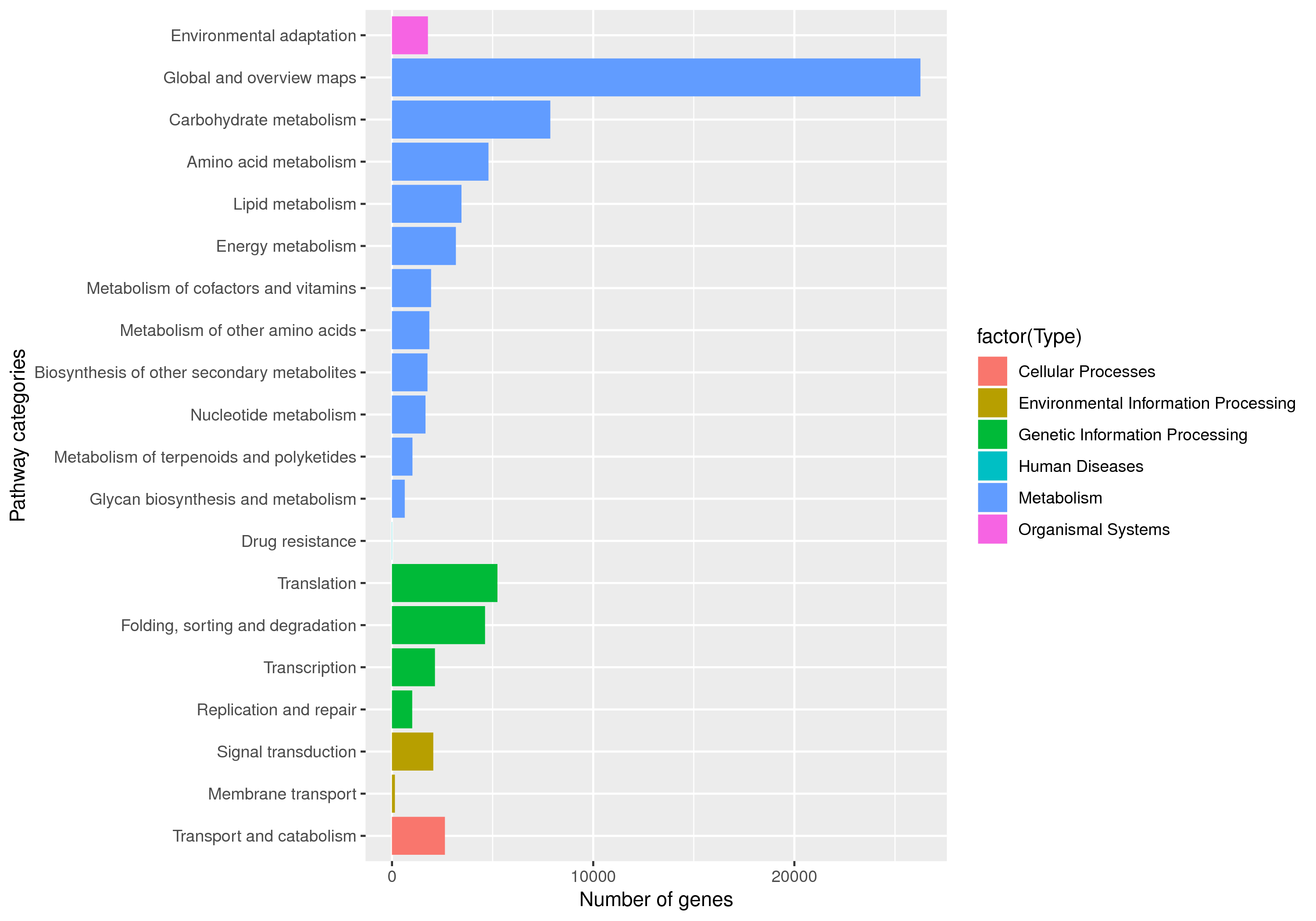
**

C

**Figure S3.** Functional annotation of unigenes annotation in three databases. Histogram presentation of GO function (**A**), COG (**B**) and KEGG (**C**) classifications of unigenes.
