# Supplementary material for: Comparative transcriptome analysis of resistant and susceptible Kentucky bluegrass varieties in response to powdery mildew infection
Source: BMC Plant Biol. 2022 Nov 2;22:509. doi: 10.1186/s12870-022-03883-4 (PMC9628184; doi:10.1186/s12870-022-03883-4)
Supplement: Supplementary file 6 — Additional file 6: Figure S4. Effect of powdery mildew infection on starch and sucrose metabolism pathway of Poa pratensis. Note: Red background indicates up-regulated expression, blue background indicates down regulated expression, and green background indicates up-regulated expression down regulated expression. [file 12870_2022_3883_MOESM6_ESM.docx]

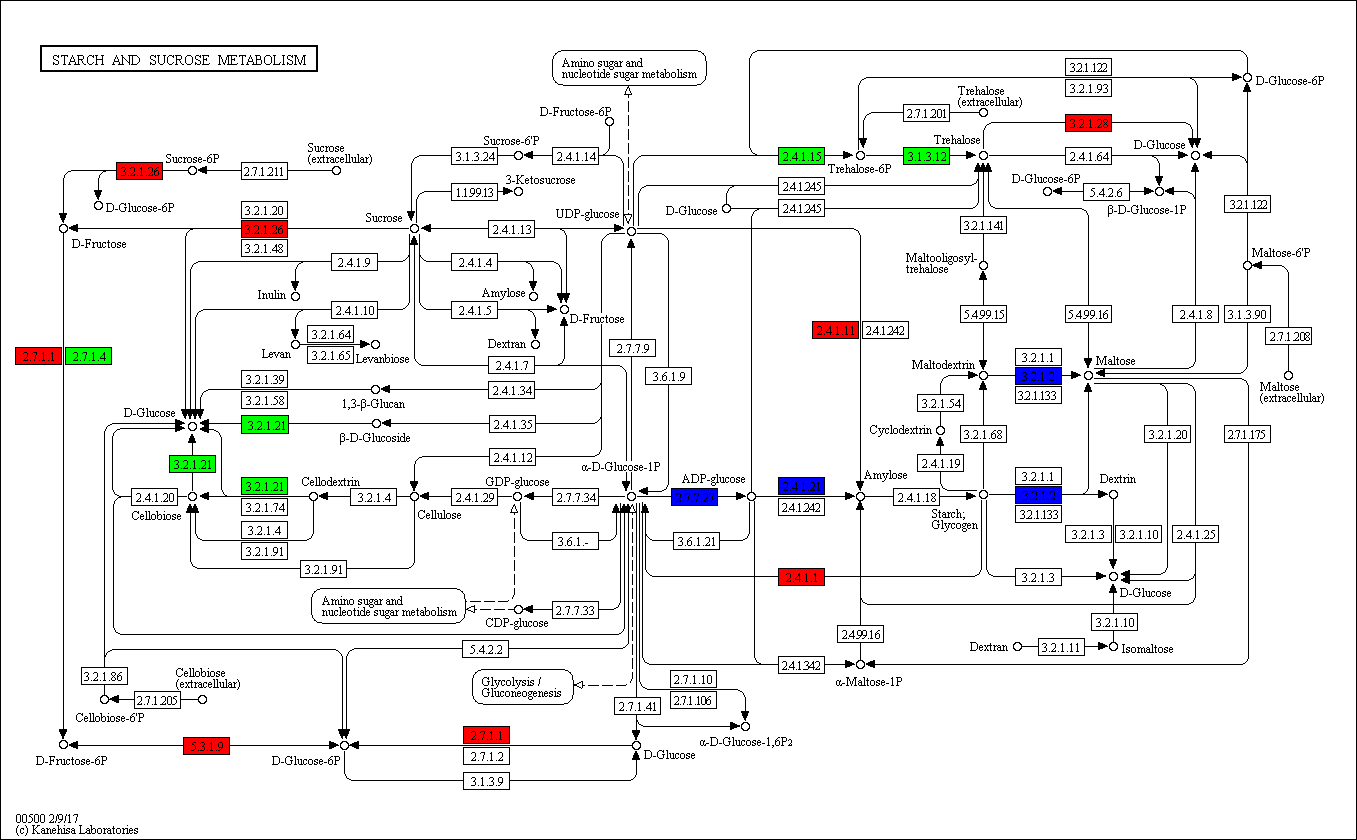


**Figure S4.** Effect of powdery mildew infection on starch and sucrose metabolism pathway of *Poa pratensis*. Note: Red background indicates up-regulated expression, blue background indicates down regulated expression, and green background indicates up-regulated expression down regulated expression.
