# Supplementary material for: Comparative transcriptome analysis of resistant and susceptible Kentucky bluegrass varieties in response to powdery mildew infection
Source: BMC Plant Biol. 2022 Nov 2;22:509. doi: 10.1186/s12870-022-03883-4 (PMC9628184; doi:10.1186/s12870-022-03883-4)
Supplement: Supplementary file 7 — Additional file 7: Table S3. The date of RNA-seq and qRT-PCR. [file 12870_2022_3883_MOESM7_ESM.docx]

**Table S3** The date of RNA-seq and qRT-PCR

| Gene ID | Log_2_ Fold Change (RNA-seq) | Log_2_ Fold Change (qPCR) |
| --- | --- | --- |
| TRINITY_DN122128_c4_g1_i5 | 3.78 | 1.23 |
| TRINITY_DN138869_c0_g15_i1 | 3.1 | 2.78 |
| TRINITY_DN141057_c8_g11_i1 | 3.47 | 2.65 |
| TRINITY_DN119283_c0_g3_i2 | 3.43 | 3.79 |
| TRINITY_DN120871_c1_g1_i5 | 4.12 | 1.14 |
| TRINITY_DN139070_c8_g16_i1 | -1.42 | -2.46 |
| TRINITY_DN112088_c3_g11_i2 | -1.08 | -1.95 |
| TRINITY_DN141119_c0_g17_i3 | -1.8 | -1.67 |
| TRINITY_DN144777_c0_g19_i1 | -1.27 | -0.77 |
| TRINITY_DN135315_c1_g2_i1 | -1.36 | -0.76 |
| TRINITY_DN137646_c2_g6_i4 | 5.47 | 3.32 |
| TRINITY_DN114396_c0_g2_i3 | 3.78 | 2.16 |
| TRINITY_DN139523_c0_g1_i1 | 3.18 | 2.78 |
| TRINITY_DN37008_c0_g1_i1 | 4.81 | 3.17 |
| TRINITY_DN139750_c1_g25_i1 | 3.88 | 2.78 |
| TRINITY_DN114281_c1_g20_i1 | -1.02 | -0.87 |
| TRINITY_DN138816_c1_g12_i1 | -1.09 | -1.78 |
| TRINITY_DN114963_c1_g2_i16 | -1.18 | -1.42 |
| TRINITY_DN93362_c1_g5_i1 | -1.04 | -1.36 |
| TRINITY_DN46679_c0_g1_i1 | -1.26 | -2.75 |
