# Supplementary material for: Comparative transcriptome analysis of resistant and susceptible Kentucky bluegrass varieties in response to powdery mildew infection
Source: BMC Plant Biol. 2022 Nov 2;22:509. doi: 10.1186/s12870-022-03883-4 (PMC9628184; doi:10.1186/s12870-022-03883-4)
Supplement: Supplementary file 8 — Additional file 8: Table S4. Primer sequence information of qRT-PCR. [file 12870_2022_3883_MOESM8_ESM.docx]

**Table S4** Primer sequence information of qRT-PCR

| Gene ID | Gene functional annotation | Primers’ sequence (forward primer/reverse primer) |
| --- | --- | --- |
| TRINITY_DN122128_c4_g1_i5 | Wall-associated receptor kinase 3 | TGGTCGGTGTATCTCCTTGGCTAC/  GACGACGAATGAAGGTAGGTGAGC |
| TRINITY_DN138869_c0_g15_i1 | 4-coumarate:coenzyme A ligase | TGGTCGGTGTATCTCCTTGGCTAC/  GACGACGAATGAAGGTAGGTGAGC |
| TRINITY_DN141057_c8_g11_i1 | Pathogenesis-related protein 10 | CACCTTGCCACCCTCCAACATC/  CACCGTGATGAAGAGCCGAGTTC |
| TRINITY_DN119283_c0_g3_i2 | UDP-glycosyltransferase UGT75E2 | AACACAAGGCTGGACACGATGC/  ACTCCAAGGACGCCGATGATAGG |
| TRINITY_DN120871_c1_g1_i5 | Flavonol synthase/flavanone 3-hydroxylase | GGCGACTACTACTTCCTCCACCTC/  CACCACCTCCTCACCGTACTCC |
| TRINITY_DN139070_c8_g16_i1 | Ribulose bisphosphate carboxylase/oxygenase activase B, chloroplastic | CACCCTGCTTCTTCACCTTCTTCC/  GTCCGATGGCTGCTGCTTTCTC |
| TRINITY_DN112088_c3_g11_i2 | Putative isoaspartyl peptidase/L-asparaginase 2 | TCGGTCGCTCACTCCCAGATG/  CGAGGTGGCGTATGGGTTTAACTG |
| TRINITY_DN141119_c0_g17_i3 | ATP sulfurylase | AAAAGACCATCTGACCGTCTCGTG/  GATGCCCGGAGGAGTGAGGAG |
| TRINITY_DN144777_c0_g19_i1 | Aminolevulinic acid dehydratase | AAGTTAGCGGGCGATATGGTTGTC/  AGACCGAAGGCACCCGAAGG |
| TRINITY_DN135315_c1_g2_i1 | Glutathione S-transferase zeta class | CGTCCGCCATCGCCAATTCC/  CGAACCCGACTGAACTGTCCTTG |
| TRINITY_DN137646_c2_g6_i4 | Sugar transport protein 1 | GCTCGCCATCTGCTTCTTCCG/  TTGTCTTCTTCACCTGCGTCATCG |
| TRINITY_DN114396_c0_g2_i3 | Putative LRR receptor-like serine/threonine-protein kinase | TCTTCTTCGCCGCAGCATTACG/  TCTCTTCTGGGCTGGCAAGGG |
| TRINITY_DN139523_c0_g1_i1 | Putative Cytochrome P450 71D11 | GCAAGCTCTGCGTCGTGGAG/  TTGGAGGCGGCGATGGAGAG |
| TRINITY_DN37008_c0_g1_i1 | Glutathione-dependent oxidoreductase | GTCCAAGAGTAGTGCAGCGTCTG/  CATGCGTCCGTGCTCTGTCG |
| TRINITY_DN139750_c1_g25_i1 | Calreticulin | GCTGACGATGAGGACAAGGATGAC/  CAATGTAGCGGCGGCCTCTTC |
| TRINITY_DN114281_c1_g20_i1 | Phenylalanine ammonia-lyase, partial | GTCAGCACCTTCTTGGACACCTG/  CCATCGACCTCCGCCACCTC |
| TRINITY_DN138816_c1_g12_i1 | Cellulose synthase A catalytic subunit 9 | ACGACGCCGATGATGTTGATGAC/  AGGTGACGAGGACGACGAGTTC |
| TRINITY_DN114963_c1_g2_i16 | Putative glutamate carboxypeptidase 2 | CATCGTCTCAGCCATCGCCAAC/  GATGCCTTGGACACAGCCTTCG |
| TRINITY_DN93362_c1_g5_i1 | Peroxisomal membrane protein 11-1 | CGTATTTGTCGGCACCCCTGAG/  TACTGCTGGATGGCGTCCTCTG |
| TRINITY_DN46679_c0_g1_i1 | Ribulose bisphosphate carboxylase activase b | TTCCGTAGGCCCTGGCTGATG/  CTTGTTACCGACATCTCCGACGAC |
